# A Study on the Analytical Sensitivity of 6 BSE Tests Used by the Canadian BSE Reference Laboratory

**DOI:** 10.1371/journal.pone.0017633

**Published:** 2011-03-11

**Authors:** John G. Gray, Sandor Dudas, Stefanie Czub

**Affiliations:** Lethbridge Laboratory, Canadian Food Inspection Agency, Lethbridge, Alberta, Canada; Creighton University, United States of America

## Abstract

*Bovine spongiform encephalopathy* (BSE) surveillance programs have been employed in numerous countries to monitor BSE prevalence and to protect animal and human health. Since 1999, the European Commission (EC) authorized the evaluation and approval of 20 molecular based tests for the rapid detection of the pathological prion protein (PrP^sc^) in BSE infection. The diagnostic sensitivity, convenience, and speed of these tests have made molecular diagnostics the preferred method for BSE surveillance. The aim of this study was to determine the analytical sensitivity of 4 commercially available BSE rapid-test kits, including the *Prionics®-Check WESTERN*, the *Prionics® Check-PrioSTRIP*™, the *BioRad® TeSeE*™ ELISA, and the *IDEXX® HerdChek*™ EIA. Performances of these tests were then compared to 2 confirmatory tests, including the *BioRad® TeSeE*™ *Western Blot* and the modified *Scrapie Associated Fibrils* (*SAF*)*/OIE Immunoblot*. One 50% w/v homogenate was made from experimentally generated C-type BSE brain tissues in ddH_2_O. Homogenates were diluted through a background of BSE-negative brainstem homogenate. Masses of both positive and negative tissues in each dilution were calculated to maintain the appropriate tissue amounts for each test platform. Specific concentrated homogenization buffer was added accordingly to maintain the correct buffer condition for each test. ELISA-based tests were evaluated using their respective software/detection platforms. Blot-protocols were evaluated by manual measurements of blot signal density. Detection limitations were determined by fitted curves intersecting the manufacturers' positive/negative criteria. The confirmatory SAF Immunoblot displayed the highest analytical sensitivity, followed by the IDEXX® *HerdChek*™ EIA, *Bio-Rad® TeSeE*™ *Western Blot*, the *Bio-Rad® TeSeE*™ ELISA, *Prionics®-Check PrioSTRIP*™, and *Prionics®-Check WESTERN*™, respectively. Although the tests performed at different levels of sensitivity, the most sensitive and least sensitive of the rapid tests were separated by 2 logs in analytical sensitivity, meeting European performance requirements. All rapid tests appear suitable for targeted BSE surveillance programs, as implemented in Canada.

## Introduction

*Bovine Spongiform Encephalopathy* (BSE) is a fatal and, thus far, untreatable neurodegenerative disease that affects a variety of mammals, including humans. Public sensitivity to BSE has become intense due to the European outbreak of *bovine spongiform encephalopathy* (BSE) in the 1980s and 1990s [1]–[4]. Ensuing cases of a variant form of Creutzfeldt-Jakob disease (vCJD) in humans began appearing, and has been largely attributed to the consumption of tainted products from BSE infected cattle [1], [2], [5]. Concerns over BSE are also exacerbated due to the obscure nature of infection and transmissibility through food consumption [5], [6]. The consumer of BSE-contaminated foods may not show clinical signs of infection until a number of years later, thus making the source of the infectious material difficult to trace. Incubation periods for disease progression can take between four to six years in cattle, and from ten to fifteen years in humans [5].

The major cause for the outbreak was the recycling of meat and bone-meal by-products (MBM) from slaughtered animals, where it was used as an additive in livestock feed to boost nutritional value. Livestock consuming the BSE-contaminated feeds would become infected and further propagate the disease upon their slaughter. As this was a cyclic process, the rate of infectious MBM entering the feed system, as well as the rate of newly infected animals, increased exponentially [4], [7].

A principle method to control the threat of BSE in human food products is the surveillance of livestock destined for slaughter and consumption. Prior to current active-surveillance programs, BSE surveillance had been done by passive-surveillance, which relied on farmers and veterinarians to visibly identify animals clinically symptomatic for BSE [4]. These animals present themselves as ataxic, or display exaggerated behaviours [8]. Once molecular diagnostic test became available, active-surveillance programs were started in Switzerland with a targeted surveillance, where cattle that were dead, down, distressed, or diseased were selected to be tested for BSE [4].

In 1999, the *European Commission* (EC) evaluated four BSE tests designed to detect the disease associated BSE prion-protein isoforms [9]. In 2001, the European Union implemented a testing regime for slaughtered cattle, recommending that over the age of 30 months be tested [3], [4], [10], [11]. By the end of 2002, two thirds of European BSE cases were discovered by rapid-tests used in active-surveillance programs [12]. Currently, commercially available BSE rapid-tests are a vital component to monitor the efficacy of a country's BSE surveillance program.

The physiological cause for BSE is attributed to the molecular state of the prion protein. The cellular form of the prion (PrP^c^) is a GPI-anchored membrane-glycoprotein, and is commonly found in many cell-types— predominantly neurons [5], [13], [14]. The disease associated prion conformer, PrP^sc^, is amyloidogenic and cytotoxic, and is believed to possess the ability to convert PrP^c^ into PrP^sc^ upon molecular contact [13], [14]. Unique physical properties of the prion protein are exploited in order to detect its presence in tissues. PrP^sc^ is more resistant to protease digestion than PrP^c^ [5], [13]–[16]. When PrP^sc^ is exposed to proteinase K (PK), western blot results show three distinct glycoforms of the protein, where each glycoform is differentiated by a quantitative ratio and molecular weight from one another. Analysis of these glycoform ratios and their respective molecular weights is use to identify the type of BSE, whether it is classical-BSE or an atypical form [15], [17]. When PrP^c^ is exposed to PK, it is mostly digested, and no PrP-relevant bands can usually be detected [15].

The PrP^sc^ isoform possesses a different tertiary structure than PrP^c^. The native PrP^c^ exists in a α-fold, where as PrP^sc^ presents itself in a β-fold [5], [14], [18]. Conformational detection technology takes advantage of this property to selectively capture PrP^sc^, without compromising PrP^sc^ presence by a potential over exposure to proteases. This is done using conformation-dependant antibodies and/or synthetic ligands [16], [19].

This aim of this study is to extend the current knowledge of BSE diagnostic tests by identifying a theoretical limit-of-detection (L.O.D.) for each of four commercially available BSE rapid-test kits, as well as two confirmatory tests. The four rapid-tests kits are the *Prionics®-Check PrioSTRIP*™, *Bio-Rad® TeSeE*™ ELISA, *Prionics*®-*Check WESTERN*™, and *IDEXX® HerdChek*™ *BSE EIA*—all of which are used for surveillance and/or disease confirmation in the Canadian BSE Reference Laboratory. The four surveillance tests have been evaluated, and approved for use, by the EC via the *European Food Safety Authority* (EFSA) [9], [20], [21].

The confirmatory tests in this study are the Canadian BSE Reference Laboratory's rendition of the *Scrapie Associated Fibril Test* (S.A.F.)/O.I.E immunoblot [22], [23], as well as the commercially available Bio-Rad® *TeSeE*™ Western Blot. The primary evaluation of these rapid-tests focused mainly on the diagnostic specificity and sensitivity on a large number of field samples [9], [20]. However, this study aims to characterize the analytical sensitivity of these tests by defining each test's detectable penultimate dilution of an initially strong BSE-positive sample. Each test was challenged with the same BSE-positive material—consistent in strain and PrP^sc^ concentration—which originated from an experimentally infected bovine sacrificed with endstage clinical disease.

By serially diluting confirmed strong BSE-positive material into a background of confirmed BSE-negative material, each test was evaluated over diminishing levels of an identical strain of BSE PrP^sc^ in a consistent background of non-infectious PrP^c^. This study aimed to characterize each test's behaviour and performance as the number of infectious BSE units (PrP^sc^) became scarcer.

Although detection limitations are outlined as per diagnostic criteria within the manufacturers' instructions, this study considered a theoretical L.O.D. for each test, based on *elevated* negative or aberrant results. Being critical of such results could help identify extremely weak positive BSE cases, based on the knowledge of each tests performance over low PrP^sc^ concentrations (analytical sensitivity).

## Materials and Methods

### Experimental BSE Infections and Homogenate Preparation

To generate the pooled experimental BSE sample for intra cranial inoculations, rostral medulla from a classical (C-type) Canadian BSE field case was homogenized in *phosphate buffered saline* (PBS) {*2.7 mM KCl; 1.5 mM K* *(PO_4_)* *; 8.1 mM Na_2_* *(PO_4_)* *; 137 mM NaCl; pH: 7.4±0.2*} to a final concentration of 10%w/v using a MediFAST/Prypcon system. Homogenates were then transferred to 2 mL microfuge tubes and centrifuged at 500rcf for 10 minutes. Supernatants were collected and transferred to 1.5 mL microfuge tubes, in 1.5 mL aliquots. Homogenates were stored at −20°C. All tissues, homogenates, and inoculum were created and stored in a biosafety level-3 (BSL-3) facility.

For intra cranial cattle inoculations (protocol #05001), the aforementioned homogenates were thawed, sonicated 3 times (30 s per sonication), then centrifuged for 10 minutes at 500rcf. Supernatants were aspirated into syringes, fitted with 16-gauge needles. Two 6 month old calves were sedated and inoculated intracerebrally with the positive inoculum through a small hole drilled in the cranium. Each animal received 1 mL (∼100 mg C-type BSE tissues) of homogenate. Animals were observed until clinical symptoms were apparent and the animal was deemed BSE-positive. Animals were euthanized, followed by post-mortem examination and tissue collection, in a BSL-3 post-mortem facility. Central nervous system tissues were collected and stored at -80°C. The experimental procedure was approved by the Burnaby-Lethbridge Animal Care Committee (BLACC), protocol #05001.

### Preparation of Diagnostic Test Homogenates

Tissues from the medulla oblongata, thalamus, and colliculus of the two inoculated calves were trimmed and confirmed for positive-reactivity using the *Prionics*® -*Check PrioSTRIP*™ (results not shown). Medulla oblongata, thalamus, and colliculus tissues were then homogenized in de-ionized water (ddH_2_O) water to a final concentration of 50%w/v and pooled. A pool of BSE negative-tissue macerate, confirmed negative by S.A.F./O.I.E. Immunoblot (results not shown), was created from brainstem material from randomly selected Canadian surveillance samples of 17 bovines. The macerate was aliquoted and stored at −80°C. A BSE-negative 50%w/v homogenate in ddH_2_O was created from the macerate. All 50%w/v homogenates were homogenized using a hand-held homogenizing unit.

### Rapid Test Setup and Execution

#### *Prionics®-Check WESTERN*™

Negative 50%w/v homogenate was homogenized using *Prionics*'® *Priogenizer* to a final concentration of 10%w/v in 1x kit homogenization buffer. Homogenate was appropriately aliquoted into a 96 sample plate. Positive 10%w/v homogenate (in 1x homogenization buffer) was added to the first well, and then serially diluted across the plate. The dilutions were loaded on to a *Prionics*®-*Check WESTERN*™ digestion plate, and the test was conducted as per the manufacturer's instructions. All 17-well, 12% SDS-PAGE pre-cast gels (Invitrogen™) were run in MOPS SDS-PAGE running buffer with antioxidant (Invitrogen™) at room temperature. Wet transfers were performed in transfer buffer (40.34 mM *glycine*; 118.98 mM *Tris*; 2.47 M *methanol*), which was continuously cooled to 4°C with a coolant-circulating system and coil. TBST buffer (2.69 mM *KCl*; 136.90 mM *NaCl*; 24.76 mM *Tris*; 0.45 mM *Tween 20*; pH: 7.4) was used to dilute the kit antibodies, and used for all washing steps. CDP-*Star*™ (Roche) was used as the chemiluminescent substrate for the alkaline-phosphatase [15], [24].

Blots were detected using x-ray film, *GBX* developer, and *GBX* fixer (Kodak). Film exposure times were assayed at 4, 8, 16, and 32 minutes; development time for all exposures was 35 seconds. All films were fixed for 3 minutes. The best balanced exposure was selected for the analysis.

#### *Prionics®-Check PrioSTRIP*™

Using the *Prionics*® *Priogenizer*, 50%w/v negative and positive homogenates where homogenized to a final 10%w/v homogenate in 1x *Prionics*® homogenization buffer. The negative homogenates were pooled and appropriately aliquoted into a 96-well sample plate. An aliquot of 10%w/v positive tissue (in 1x *Prionics*® -*Check PrioSTRIP*™ homogenization buffer) was added to the first well of the plate, and serially diluted across the plate in 10%w/v negative homogenates. Diluted positive samples were then loaded on a digestion-plate, and the test was executed as per the *Prionics*® -*Check PrioSTRIP*™ package insert [25]. Combs were then scanned using the appropriate scanner (Perfection V700 Photo, Epson) and software (*PrioSCAN*™ v3.0) provided by *Prionics*®.

#### *Bio-Rad® TeSeE*™ ELISA

To account for excess ddH_2_O in 50%w/v negative and positive homogenates, buffer within the kit tissue grinding tubes was lyophilized using a SpeedVac® System (Thermo Savant) in order to concentrate the grinding buffer (45°C x 5.1 inHg x 3 h). Negative 50%w/v homogenate was added to the tubes (350 mg tissue mass); one tube was loaded with 50%w/v positive homogenate (350 mg tissue mass). DdH_2_O was added to adjust the tissue concentration to 25%w/v, as required by the kit protocol. Samples were again homogenized, using the *TeSeE*™ *Precess 48* homogenizing system [26].

Negative homogenates were diluted to 25%w/v and pooled. The positive homogenate was serially diluted throughout the negative homogenates. The dilutions were aliquoted (250 µL) into 2 mL microfuge tubes, and the test was performed as per the manufacturer's manual method instructions. All wash steps were conducted by automated PW40 plate washers (*Bio-Rad*® Laboratories), as supplied by *Bio-Rad*® Laboratories. ELISA plates were analyzed using the Model 680TSE microplate reader (*Bio-Rad*® Laboratories) and affiliated software [26].

#### *IDEXX® HerdChek*™ *(Bovine Spongiform Encephalopathy Antigen Test Kit, EIA)*

Sample grinding tubes were lyophilized using a *SpeedVac®* System (Thermo) to concentrate the grinding buffer (45°C x 5.1inHg x 3 h). 600 µL of 50%w/v negative homogenate (300 mg tissue mass) was distributed into the lyophilized buffer tubes. DdH_2_O was used to restore the tubes to the original tube buffer volume, bringing the homogenate to a 1x buffer environment. The BSE positive tube was prepared in an identical manner.

All negative tubes were pooled and redistributed in the appropriate aliquots. The BSE positive homogenate was serially diluted across the negative tubes. The remainder of the test was continued as per the manufacturer's instructions. All wash steps were done by automated plate washers. The antigen capture plate was analyzed using a *Bio Tek ELX 800* microplate reader, supplied by IDEXX® Laboratories [27].

### Confirmatory Test Setup and Execution

#### *Bio-Rad® TeSeE*™ *Western Blot*

Preparation of negative homogenates and positive dilutions were prepared identically to those previously described for the *TeSeE*™ ELISA. Sample dilutions were processed as per the manufacturer's instructions, using the equipment recommended by *Bio-Rad*® Laboratories. All Bio*-Rad® Criterion*™ 12% SDS-PAGE gels were run at room temperature; transfers were continuously cooled to 4°C with a circulating cooling-coil. Millipore® Immobilon™ Western HRP substrate was used as the test's chemiluminescent substrate [28].

Blots were detected with x-ray film, GBX developer, and GBX fixer (Kodak). Films were exposed in the following intervals: 10 s, 20 s, 30 s, 1 m, and 2 m. Films were developed for 3 s, and fixed for 3 m. The best balanced exposure was selected for analysis.

#### *Scrapie Associated Fibril (S.A.F.)/O.I.E. Immunoblot (Confirmatory Test)*

Positive and negative 50%w/v homogenates were resuspended to 25%w/v in brain lysis buffer (BLB) (10 g *sodium N-laurylsarcosine*; 100 mL, 0.01 M *sodium phosphate*; pH 7.4) (Sigma-Aldrich) with 100 µL 100 mM NEM (0.313 g *N-ethyl-maleimide*; 25 mL *1-propanol*) (Sigma-Aldrich), and 100 µL 100 mM PMSF (0.435 g, *phenylmethylsulfonyl fluoride*; 25 mL, *1-propanol*) (Sigma-Aldrich). Samples were homogenized using a MediFAST/Prypcon system. Three drops of *1-octanol* (Sigma-Aldrich) was added to each homogenate to reduce the amount of froth generated by homogenization. Negative homogenates were pooled, and aliquoted into 15 mL tubes. The positive homogenate was serially diluted along the negative homogenates [22], [23].

Diluted samples were transferred into Quick-Seal tubes (Beckman-Coulter) using a syringe and cannula. Tubes were balanced, heat-sealed, and loaded into a 70Ti ultracentrifuge rotor (Beckman-Coulter). The lysates were then centrifuged (20,000rcf×30 min; 10°C). The supernatant was collected and transferred to new Quick-Seal tubes. The tubes were balanced with BLB, heat-sealed, and centrifuged again (177,000rcf x 2 h15 min; 10°C). Pellets were vigorously resuspended in 1.5 mL ddH_2_O plus 25 µL 1 M Tris-HCl. Samples were incubated in a water-bath at 37°C for 15 m [22], [23].

3 mL of 15% KI-HSB (60.4 mM *sodium thiosulphate pentahydrate*; 36.8 mM *N-lauroylsarcosine*; 100 mM *Tris-HCl* [pH 7.4]; 903.6 mM *potassium iodide*) was added to the samples, and incubated in a water-bath at 37°C for 30 min, with occasional mixing. Proteinase K (Roche) was added to each dilution to a final concentration of 10 µg/mL. Tubes were then incubated in a water-bath at 37°C for 1 h. 4.5 mL of 10% KI-HSB (60.4 mM *sodium thiosulphate pentahydrate*; 36.8 mM *N-lauroylsarcosine*; 100 mM *Tris-HCl* [pH 7.4]; 602.4 mM *potassium iodide*) was added to the samples. Samples were transferred to Quick-Seal tubes [23].

2 mL of a 20% *sucrose* cushion (20 g *sucrose*; 80 mL 10% KI-HSB) was carefully deposited to the bottom of the tubes containing the digested samples. Tubes were filled and balanced with 10% KI-HSB, heat-sealed, and centrifuged at 189,000rcf for 1 h at 10°C. The supernatant was discarded, and the resulting pellets were resuspended in 40 µL of *Prionics®* PAGE sample buffer. Samples were transferred to 2 mL microfuge tubes and sonicated for 30 s, using Virsonic 550 sonicator [23].

Samples were heated at 95°C for 5 m, and loaded onto a 12-well, 12% SDS-PAGE gel (Invitrogen™). The remainder of the blotting protocol was based on the *Prionics®*-*Check WESTERN*™ rapid-test [15], [23], [24].

### Test Performance Evaluation

The unit of measurement used to evaluate the analytical test sensitivity was a tissue-ratio of milligrams negative-tissue to milligrams positive-tissue (mg-/mg+). For test platforms requiring software-based result interpretation, the software output (positive or negative) was deemed the final result for the manufacturers' criteria (*Bio-Rad*® *TeSeE*™ ELISA, IDEXX® *HerdChek*™ *BSE EIA*, *and Prionics*®-*Check PrioSTRIP*™). Output values were plotted to attain a performance curve for each test. Data were fitted with a saturative, total binding function, accounting for the total multitude of binding components, as well as any unforeseen non-specific factors: 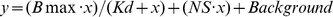 (*PRISM* v5.0). A coefficient of determination,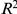, was determined for each curve to support the tests' consistency and performance. 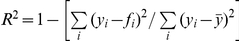, where 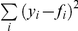 is the sum of squares of the data residuals, and 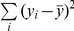
is the total sum of squares of the data (*PRISM v5.0*).

For western-blot protocols (*Prionics*®-*Check WESTERN™*, *Bio-Rad*® *TeSeE Western Blot*, *S.A.F./O.I.E. Immunoblot*), blot films were analyzed using ChemiDoc XRS (*Bio-Rad*® Laboratories). Manual measurements of PrP^sc^ glycoform band-densities (intensity x mm^2^) were conducted, and used as a physical property to measure PrP^sc^/antibody binding. A total-binding function (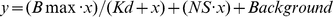) for the data was plotted (*PRISM* v5.0.). 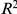 values were also determined for the fitted curves.

Our definition of a false-negative result pertains to a sample which has been included in a BSE-positive material dilution series that had generated a result indistinguishable from that of a known BSE-negative sample. The theoretical L.O.D. refers to the limit for the penultimate dilution for the test being challenged. This was identified by visually inspecting the disappearance of bands, as was as the point the binding curve for the diglycosylated PrP^sc^ band intersected the background density of the particular blot. For ELISA based protocols, this limit was determined when the binding curve intersected the tests' mean negative result values.

## Results

### Rapid Tests

#### *Prionics®-Check WESTERN*™

Prionics®-*Check WESTERN*™ diagnostic criteria for positive results pertain a visible, three band (signal) pattern, decreasing in intensity from top to bottom, where the top-band (diglycosylated band) is situated immediately following the PK-band. Weak-positive criteria consist of the presence of the top-band, where the distance between the top-band and the PK-band is more noticeable than usual, as well as lacking the presence of the second (monoglycosylated) and third (unglycosylated) bands. Negative results are only lanes without bands (other than the PK-band) and the kit's positive control is clearly visible [15], [24].

**Figure 1:**
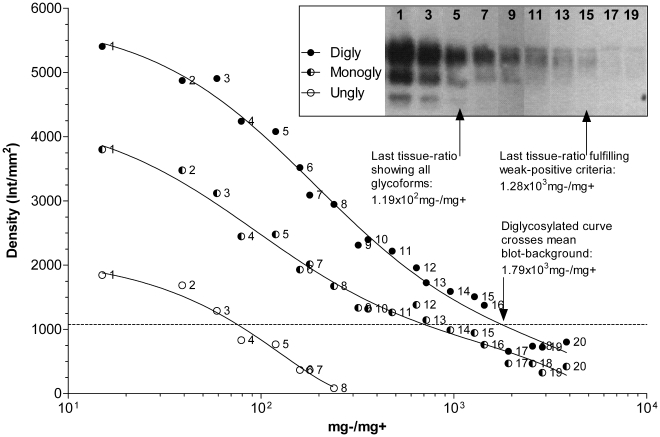
The curves depict densities of PrP^sc^ bands (diglycosylated (Digly), monoglycosylated (Monogly), unglycosylated (Ungly)) in the Prionics®-*Check WESTERN*™ western blot. Numbers on the right of each density data point correspond to the numbers in the blot photo (inset). Only odd numbered data points appear in the blot photo. Each label (Digly, Monogly, Ungly) in the inset photo appears to the left of the band it describes. Inset text and arrows indicate detection limitations. *R* ^2^: Digly = 0.9870; Monogly = 0.9841; Ungly = 0.9635.

Figure 1 demonstrates that the kit was able to detect as per the positive criteria to a tissue-ratio of 1.19×10^2 ^mg−/mg+, where all glycoform bands were clearly distinguishable from the blot background. However, the weak-positive could be fulfilled up to 1.28×10^3^ mg−/mg+, where the top-band was visible just below the PK-band and no second or third band was present. At 1.79×10^3^ mg−/mg+, the fitted-curve suggests the result for a weak positive sample would be indistinguishable from a negative sample, and is therefore considered the theoretical L.O.D.

Given the semi-subjective nature of western blot interpretation, individual techniques, reagents and equipment used, the theoretical L.O.D. for blot protocols will likely have a greater degree of variability within detection margins as compared to the other ELISA-based rapid-tests. The margin of sensitivity between the Prionics®-*Check WESTERN*™ and the Bio-Rad™ *TeSeE*™ ELISA kits is likely narrower than what is reported in this study. The Prionics®-*Check WESTERN*™ kit was found to be 10 fold more sensitive if one considers the weak-positive criteria to be the detection limit, rather than the requirement for all PrP^sc^ glycoforms to be present.

#### *Prionics®-Check PrioSTRIP*™

**Figure 2:**
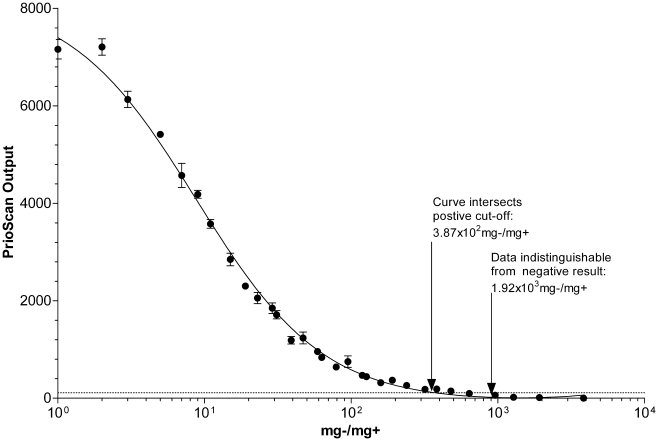
The Prionics®-*Check PrioSTRIP*™ output curve plotted against PrP^sc^ content in the respective tissue homogenates. Inset text and arrows indicates detection limitations. *R* ^2^ = 0.9870.

In figure 2, the *Prionics®-Check PrioSTRIP*™ accurately, and consistently, detected a tissue-ratio of 3.87×10^2^ mg−/mg+ as positive above the particular kit-lot's cut-off value. Non-zero results could be detected up to—but not past—1.92×10^3 ^mg−/mg+. This value was the identified theoretical L.O.D. Because zero is the inarguable negative result, any value above zero could be considered suspicious. If non-zero results are treated as initial-reactor samples, the Prionics®-*Check PrioSTRIP*™ is within the same log-base of detection as the Prionics® -*Check WESTERN*™'s weak-positive criteria and the Bio-Rad™ *TeSeE*™ ELISA.

#### *Bio-Rad® TeSeE*™ ELISA

**Figure 3:**
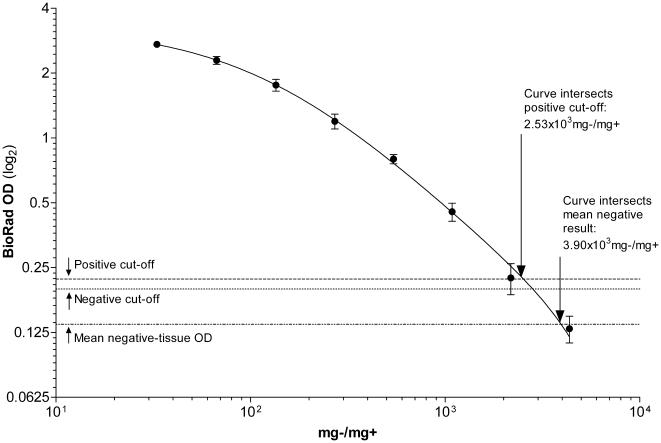
The OD curve for the *Bio-Rad*® *TeSeE* ™ ELISA plotted against PrP^sc^ content in the respective tissue homogenates. Inset text and arrows indicates detection limitations. *R* ^2^ = 0.9945.

The *TeSeE*™ ELISA correctly identified positive tissues up to a tissue-ratio of 2.18×10^3^ mg−/mg+ (*figure 3*). Although, one dilution did record in between the positive and negative cut-off criteria (inconclusive), and another dilution recorded below the negative cut-off. This dilution set was very close to the curve-derived maximal sensitivity regarding the tests' cut-off criteria of ∼2.5×10^3^ mg−/mg+. Beyond 3.90×10^3^ mg−/mg+, weak-positive dilutions were indistinguishable from negative results. This tissue-ratio was taken as the theoretical L.O.D.

The kit negative control serves to evaluate the performance of the reagents, and did not reflect the OD value of the negative tissue stock used in the experiment. The mean OD result of a negative tissue sample was 10 fold greater than the supplied kit negative. A confirmed negative tissue control would provide additional support regarding a base-line OD reference point for diagnostic samples with elevated negative OD values. Because the L.O.D. determined in this study was similar to those published in a previous study [10], these results suggest the lyophilization of the grinding buffer did not interfere with the *TeSeE*™ ELISA's performance.

#### *IDEXX® HerdChek*™ *BSE EIA*

**Figure 4:**
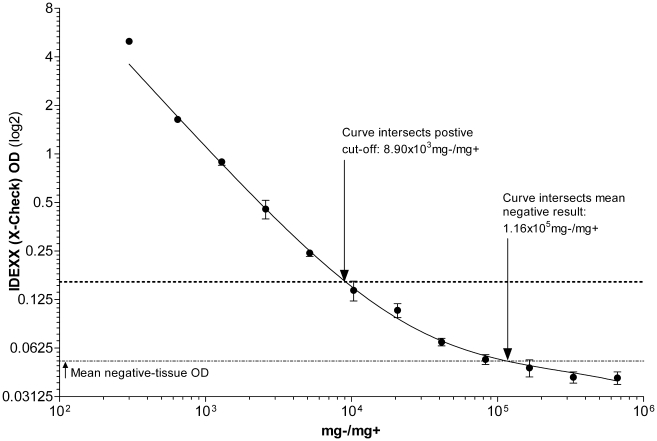
*IDEXX HerdCheck*™ *BSE EIA* OD curve plotted against PrP^sc^ content in the respective tissue homogenates. Inset text and arrows indicates detection limitations. *R* ^2^ = 0.9980.

Our results indicate that the *HerdChek*™ possesses a greater sensitivity for the BSE (C-type) tissues than the other rapid-tests. A tissue-ratio of 5.17×10^3^ mg−/mg+ was consistently positive, where the preceding tissue ratio of 1.03×10^4^ mg−/mg+ had generated both positive and negative results. The fitted-curve in *figure 4* indicates the manufacturer's cut-off criterion was met at 8.90×10^3^ mg−/mg+. The *HerdChek*™ reported at least a full log_10_ base greater sensitivity compared to the theoretical L.O.D. for the Prionics®-*Check WESTERN*™, Prionics®-*Check PrioSTRIP*™, and Bio-Rad® *TeSeE*™ ELISA.

Significantly elevated OD values of false-negative results were present up to a tissue-ratio of ∼3.00×10^4^ mg−/mg+. Elevated OD values for false negatives had a range from 0.163 and 0.06, over which had a 10-fold difference in infectious unit concentration. Because of the *HerdChek*™ *BSE EIA*'s pseudo-sigmoidal appearance on the plotted log_2_-log_10_ graph, elevated OD values below the negative cut-off are likely to be more consistently elevated on test-repeats.

The fitted-curve suggests weak-positive tissue-ratios of 1.16×10^5^mg−/mg+, and greater, are indistinguishable from negative-tissue results. This value was identified at the theoretical L.O.D.

As with the *TeSeE*™ ELISA, confirmed negative tissue samples should be tested alongside diagnostic samples to verify a base-line for true negative tissues. Because of the Seprion technology dependence on PrP^sc^ conformation/tertiary structure for detection, the test's performance regarding atypical forms of BSE may differ in sensitivity as compared to the results within this study [16], [18], [19], [29].

### Confirmatory Tests

#### *Bio-Rad® TeSeE*™ *Western Blot*

**Figure 5:**
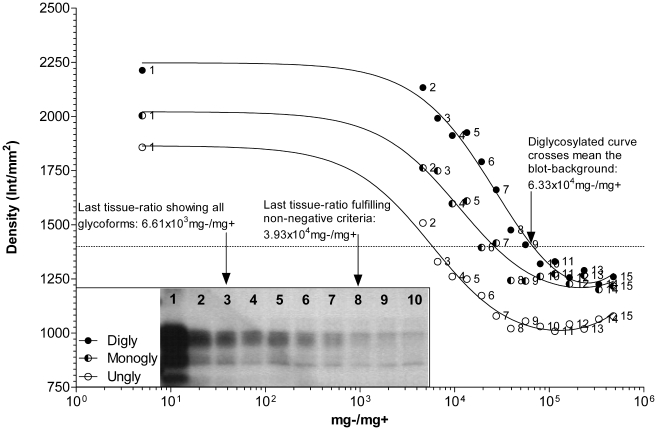
The curves depict densities of PrP^sc^ bands (diglycosylated (Digly), monoglycosylated (Monogly), unglycosylated (Ungly)) in the *Bio-Rad*® *TeSeE* ™ Western Blot. (inset) and detection curves for digly-, monogly-, and unglycosylated bands of PrP^sc^. Numbers on the right of each density data point correspond to the numbers in the blot photo (inset). Each label (Digly, Monogly, Ungly) in the inset photo appears to the left of the band it describes. *R* ^2^: Digly = 0.9858; Monogly = 0.9659; Ungly = 0.9857.

The performance of the Bio-Rad® *TeSeE*™ Western Blot, shown in figure 5, was highly sensitive—especially considering the sample purification is supported to be that of the *TeSeE*™ ELISA (Bio-Rad, personal communication), which is followed by a basic western blot protocol. Glycoforms were distinguishable up to a tissue ratio of 6.61×10^3^ mg−/mg+. Non-negative results, or only diglycosylated bands, were clearly visible up to 3.93×10^4 ^mg−/mg+. A curve-derived theoretical L.O.D. could be considered viable up to 6.63×10^4 ^mg−/mg, as this was the corresponding tissue-ratio at which the diglycosylated band's density curve intersects the mean background of the blot.

It should be noted that a band remained in the monoglycosylated region of the lane, as the density of the diglycosylated band decreased into the blot's background. As a comparative note, this behaviour parallels the weak-positive criteria outlined for the *Prionics*®-*Check WESTERN*™ test [15], [24]. This is likely due to an increased activity of PK on the minute proportion of PrP^sc^ in these high dilutions. Increased PK activity would over-digest the PrP^sc^ in the homogenate, thus resulting in obscure, depleted PrP^sc^ fragments. Smaller diglycosylated PrP^sc^ peptides may be presenting themselves in a weight range between that of the monoglycosylated and diglycosylated PrP^sc^ glycoforms, thereby diluting the signal within the expected ∼30 kDa range. However, for the purpose of this study, the disappearance of the typical prion blot signal within the diglycosylated band's weight range was considered to be the detection limit for the test, despite the presence of other bands in the lane.

#### S.A.F./O.I.E. Immunoblot

**Figure 6:**
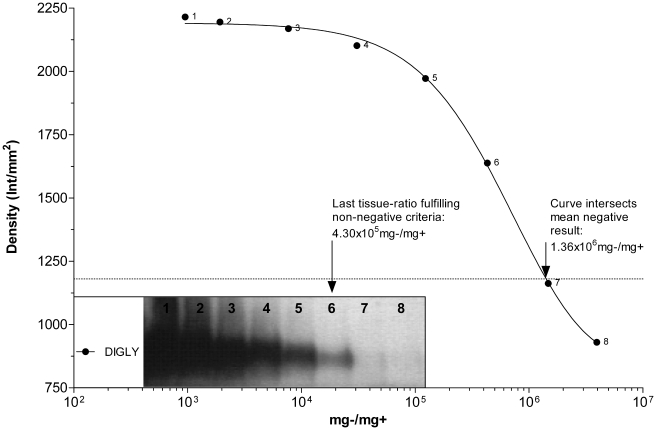
The curve depicts densities of PrP^sc^ bands in the *Scrapie Associated Fibrils* (*S.A.F.*)*/O.I.E. Immunoblot* plotted against PrP^sc^ content in the respective tissue homogenates. Numbers on the right of each density data point correspond to the numbers in the blot photo (inset). *R* ^2^ = 0.999.

Figure 6 illustrates it was possible for the S.A.F. to detect a PK resistant, ∼30 kDa band at a tissue ratio of 4.30×10^5 ^mg−/mg+. The theoretical detection limit determined by the fitted-curve was 1.36×10^6 ^mg−/mg+, where the mean blot background signal was intersected. The purification method within the S.A.F./O.I.E immunoblot protocol enhanced the standard performance of the Prionics® -*Check WESTERN*™ blot by ∼130 fold, for a non-negative sample. This method remains the most sensitive diagnostic test, clearly exceeding the performance of the second confirmatory test in this comparison, as well as the most sensitive surveillance test.

## Discussion

Because PrP^sc^ is an amyloidogenic protein [14], fibrils tend to adhere to one another in solution, potentially compromising true homogeneous serial dilutions. Given the fact that all 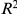 for each analytical curve was ≥0.96, and all error margins between replicates—especially for the ELISA based tests—were quite tight, this suggests that the data was fit with an appropriate function, and that the PrP^sc^ distribution amongst the serial dilutions was fair and homogeneous, and any potential post-homogenization aggregative activity between PrP^sc^ fibrils affecting dilution integrity was negligible. Slightly lower 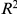
values for the western blot data are likely attributable to the nature of the manual measurements as opposed to the automated measurements from ELISA plate readers.

Diagnosing BSE via western blotting is a well characterized method; however, samples with very low PrP^sc^ concentrations show abnormal banding profiles that do not coincide with the typical criteria required for a positive diagnosis. A signal for a weak positive-sample may be given, however the sample could be diagnosed as negative, if it does not display the anticipated PrP^sc^ banding profile. For example, it could be questionable, to some, whether the diglycosylated band in tissue-ratio #15 (1.28×10^3 ^mg−/mg+) in *figure 1* is positive. However, if this sample would be tested on the *TeSeE*™ ELISA, the curve in figure 3 clearly shows the sample would register above the cut-off limit, and would label it a positive result.

The Prionics®-*Check WESTERN*™ is both a qualitative and quantitative test, as BSE-types can be distinguished by molecular weights and glycoform ratios [15], [17], [22]. It needs to be considered that a sample on an SDS-PAGE is separated into the three PrP^sc^ glycoforms from one another, thus dispersing the signal from all PrP^sc^ in the lane, rather than keeping the signal concentrated in one location. For example, the PrP^sc^ glycoform ratio for C-type BSE is approximately 68% diglycosylated PrP^sc^, 24% monoglycosylated PrP^sc^, and 8% unglycosylated PrP^sc^ [17]. This suggests the blot signal from tissue-ratio #15 (*figure* 1) only represents approximately 68% of the total prion protein in the lane.

Our results showed the Prionics®-*Check PrioSTRIP*™ performed similarly to the Bio-Rad® *TeSeE*™ ELISA. The Prionics®-*Check PrioSTRIP*™ is purely a quantitative test—all three prion-protein glycoforms are concentrated on one area of the immunochromatographic strip, the same way ELISAs concentrate the target protein on the bottom of the well. Although the Bio-Rad® *TeSeE*™ and IDEXX® *HerdChek*™ ELISAs use of colorimetric methods for detection might seem more reliable, the combination of methods and materials used in the Prionics®-*Check PrioSTRIP*™ was able to compete within a 2 log_10_ range of these tests.

Recently, there has been some controversy regarding the analytical sensitivity, performance, and consistency of the Prionics®-*Check PrioSTRIP*™, as compared to other rapid-tests [20], [21]. Although, result output values for tissue-ratios are likely to vary between kit lots, the reproducibility of results observed within this study suggests the test platform is respectably consistent, as demonstrated by the narrow standard error bars seen within *figure* 2. The sigmoidal nature of the curve, the high reproducibility, and *R^2^* value near 1 depicts the test's reliability, even at lower concentrations of PrP^sc^ in infected tissues.

Our study's Prionics®-*Check PrioSTRIP*™ results are only based on the use of the computerized scanning method/software, and not on the visual manual-reading method. Manual interpretation of the combs, although an approved method of interpreting results [25], would not have served well at low positive tissue concentrations. Visual perceptions and personal bias between individual readers is likely more subjective than an electronic-based system, where bias is eliminated. This study would always support the use of the appropriate scanning software and equipment, as suggested by Prionics®.

The ideal performance for a BSE rapid-test kit would demonstrate a sigmoid-type detection curve, plotted on a log_x_-log_y_ graph. Such a test would likely perform more consistently at lower concentrations of PrP^sc^, rather than test platforms with detection curves decreasing hyperbolically over decreasing PrP^sc^ concentrations. For example, the tissue-ratio at the *HerdChek*™'s cut-off was 8.9×10^3 ^mg−/mg+, and would generate an OD value of ∼0.163. A 2-fold less concentrated sample (∼1.78×10^4 ^mg−/mg+) would generate an OD result of ∼0.104. Although this OD value is below the cut-off criteria, it is still ∼2-fold greater than the mean negative-tissue result. In contrast, the *Bio-Rad® TeSeE*™ ELISA's cut-off OD value of 0.221 corresponds to a tissue-ratio of ∼2.53×10^3^. A 2-fold less concentrated sample would generate an extrapolated result of 0.082, more than 2.5 fold lower than the cut-off value, and lower than the mean negative tissue OD of 0.137.

In the aforementioned case, the *TeSeE*™ ELISA does not have as much tolerance for samples reading between the positive cut-off and the mean OD for a set of negative sample controls, as compared to the *HerdChek*™'s tolerance for such samples. This suggests a weak-positive sample tested on a platform like the *HerdChek*™ may stand a better chance of remaining distinguishable from the mean negative-sample result, despite potentially being labelled as “negative” by the *X-Chek*™ software. Of course, this is provided the test diagnostician was critical of each OD value registering negative, and comparing them to a mean OD from confirmed negative-tissues.

**Figure 7:**
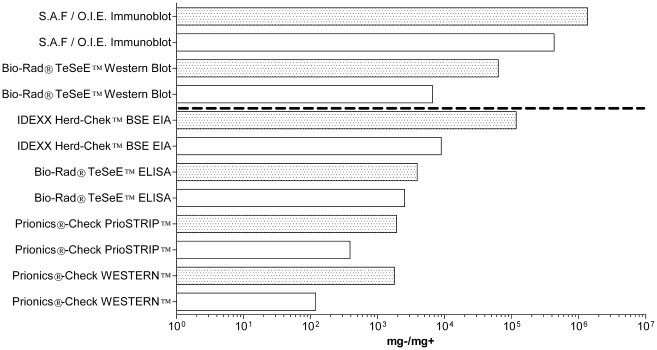
A performance summary of both rapid and confirmatory tests. White bars represent the test's detection limitations, as established by the manufacturer. Dotted bars represent our determined theoretical limit of detection for each test, where the corresponding positive tissue dilution yielded a result no different from true negative tissue. Confirmatory tests are above the dashed line. Rapid-tests are below the dashed line.

*Figure 7* depicts the relative performance of each test when the manufacturers' positive/negative cut-off diagnostic definitions were respected, as well as the L.O.D we have determined for each test, being when each test failed to generate any distinguishing results that could differ from a weak-positive sample from a true negative-sample.

In conclusion, all BSE rapid screening tests evaluated in this study are EU/EFSA approved. The results presented within this study are not preferentially condoning the use of one test over another. All rapid-tests were able to perform within a 2 log_10_ range of one another, and all coincide with EC No. 999/2001 regulations on monitoring BSE prevalence [11]—despite which diagnostic criteria was used to define the tests' L.O.D. All tests are well suited for use in targeted surveillance programs, where animals selected for BSE surveillance are typically exhibiting suspicious signs of clinical disease. Samples from animals exhibiting even subtle signs of BSE are most likely to behave as field samples used in validation exercises used for test approval.. This study elucidates the relative performance of these tests solely on experimentally generated C-Type BSE, serially diluted in known BSE-negative tissue homogenate, and not necessarily on C-type BSE occurring by natural means. No results in this study pertain to test performance regarding atypical-BSE. Field cases still have to be given the benefit of the doubt regarding the tests' true performances. Nonetheless, the presented results are meant to serve as insight into the test's performances on samples which are weak in BSE-positive PrP^sc^ units.
